# Comparison of the Concordance of Cardiometabolic Diseases and Physical and Laboratory Examination Findings between Monozygotic and Dizygotic Korean Adult Twins: A Cross-Sectional Study Using KoGES HTS Data

**DOI:** 10.3390/nu14224834

**Published:** 2022-11-15

**Authors:** Ho Suk Kang, So Young Kim, Hyo Geun Choi, Hyun Lim, Joo-Hee Kim, Ji Hee Kim, Seong-Jin Cho, Eun Sook Nam, Kyueng-Whan Min, Ha Young Park, Nan Young Kim, Younghee Choi, Mi Jung Kwon

**Affiliations:** 1 Division of Gastroenterology, Department of Internal Medicine, Hallym University Sacred Heart Hospital, Hallym University College of Medicine, Anyang 14068, Republic of Korea; 2 Department of Otorhinolaryngology-Head & Neck Surgery, CHA Bundang Medical Center, CHA University College of Medicine, Seongnam 13488, Republic of Korea; 3 Department of Otorhinolaryngology-Head & Neck Surgery, Hallym University Sacred Heart Hospital, Hallym University College of Medicine, Anyang 14068, Republic of Korea; 4 Division of Pulmonary, Allergy, and Critical Care Medicine, Department of Medicine, Hallym University Sacred Heart Hospital, Hallym University College of Medicine, Anyang 14068, Republic of Korea; 5 Department of Neurosurgery, Hallym University Sacred Heart Hospital, Hallym University College of Medicine, Anyang 14068, Republic of Korea; 6 Department of Pathology, Kangdong Sacred Heart Hospital, Hallym University College of Medicine, Seoul 05355, Republic of Korea; 7 Department of Pathology, Hanyang University Guri Hospital, Hanyang University College of Medicine, Guri 11923, Republic of Korea; 8 Department of Pathology, Busan Paik Hospital, Inje University College of Medicine, Busan 47392, Republic of Korea; 9 Hallym Institute of Translational Genomics and Bioinformatics, Hallym University Medical Center, Anyang 14068, Republic of Korea; 10 Department of Pathology, Hallym University Dongtan Sacred Heart Hospital, Hallym University College of Medicine, Hwaseong 18450, Republic of Korea; 11 Research Insititute for Complementary & Alternative Medicine, Hallym University, Hwaseong 18450, Republic of Korea; 12 Department of Pathology, Hallym University Sacred Heart Hospital, Hallym University College of Medicine, Anyang 14068, Republic of Korea

**Keywords:** dizygotic twins, monozygotic twins, cardiometabolic disease, genetic factors, environmental factors

## Abstract

This study investigated the contribution of genetic and environmental factors to cardiometabolic diseases (CMDs) by comparing disease concordance in monozygotic and dizygotic twins. This cross-sectional study analyzed 1294 (1040 monozygotic and 254 dizygotic) twin pairs (>20 years) based on the Korean Genome and Epidemiology Study data (2005–2014). The odds ratios of disease concordance were calculated using binomial and multinomial logistic regression models. The occurrence of CMDs (hypertension, hyperlipidemia, type 2 diabetes, cerebral stroke, transient ischemic attack, and ischemic heart disease) and related physical and laboratory levels did not differ between the monozygotic and dizygotic twin groups. The odds for concordance of the presence/absence of CMDs and the likelihood of incident CMD within monozygotic twins were comparable to that of dizygotic twins. The absolute differences in hemoglobin A1c, insulin, low- and high-density lipoprotein cholesterol, total cholesterol, triglycerides, and systolic blood pressure were lower in monozygotic twins than in dizygotic twins. Absolute differences in fasting glucose and diastolic blood pressure did not differ between groups. Although baseline levels of several laboratory parameters related to CMD showed a strong likelihood of heritability in monozygotic twins, CMD phenotype appears to be largely affected by environmental factors.

## 1. Introduction

Cardiometabolic disease is the collective term for the wide spectrum of metabolic abnormalities; however, it does not always begin with insulin resistance and progress to metabolic syndrome, which are characterized by hyperglycemia, dyslipidemia, obesity, and hypertension, and eventually leads to further deterioration in health, including cardiovascular disease and type 2 diabetes [1,2]. The scale of complications from these diseases may span the whole body, resulting in disabilities and morbidities, including skin ulceration, accelerated arthritis, diabetic retinopathy, reproductive organ or liver dysfunction, renal failure, and cognitive and mental disorders [3,4,5,6,7,8]. The most serious of these conditions may raise the risk of premature death from any cardiovascular disease cause, accounting for approximately one-third of all mortality [9]. The incidence of cardiometabolic diseases has been steadily increasing worldwide [9,10], with a remarkable increase from 24.9% to 31.3% in Korea between 1998 and 2007 [11]. This prevalence is relatively high compared to those of other Asian countries, including Japan (16.5% in 2004) [12], Taiwan (25.5% in 2008) [13], and China (21.3% in 2009) [14]. In Korea, in 2015, the prevalence of diabetes mellitus and cardiovascular disease was 100.01 and 101.11 per 1000 individuals, respectively, and these rates are comparable to that of the United States [9,10]. Therefore, these cardiometabolic diseases have become important public health concerns that need urgent action to achieve the public health goal of disease prevention [9,15].

Cardiometabolic diseases are closely interrelated and are considered multifaceted, involving interactions among genetic and environmental elements [1,2,5,16]. Meta-analyses of genome-wide association studies have identified multiple predisposing genetic factors from a large list of 123 candidate genes for cardiometabolic diseases [17]. Epidemiologic studies, including twin studies, have estimated the possible individual heritability of 30–60% for coronary artery diseases [18], 26–69% for type 2 diabetes [19,20,21], 24–37% for hypertension [22], 40–70% for obesity [23], and 58–66% for dyslipidemia [24,25]. An unhealthy diet, immoderate alcohol drinking, smoking, lack of physical activity, and poverty may drive the development of phenotypic variance [1,2,5]. In this context, subclinical laboratory changes in cardiometabolic risk factors are clinically important intermediate dysmetabolic processes before overt cardiometabolic disease [26,27], where lifestyle modifications may contribute to prevention [10,28]. However, the types of metabolic abnormalities in the cardiometabolic spectra that could be improved by environmental factor modifications and the degree to which they could be improved are yet to be evaluated.

Twin studies can provide valuable insights into the relative significance of genetic and environmental factors in human disorders and complicated traits because twins share both genetic resemblance and the same rearing environment [19]. Mainly, monozygotic twin cohorts with a common genotype have been used to inspect the role of environmental factors in determining complex diseases and phenotypes [29]. A greater correspondence between monozygotic twin pairs than between dizygotic ones may be ascribed to genetic factors. In contrast, a comparable magnitude of difference between monozygotic and dizygotic twin groups may be attributed to environmental influence [19]. However, few studies have fully investigated the potential genetic and environmental relevance of subclinical laboratory changes and cardiometabolic disease phenotypes in a validated twin study, with only one twin study based on the Hungarian population [30]; previous twin studies have only analyzed each abnormality among various cardiometabolic diseases, and the results have been controversial [20,21,22,31,32,33,34]. Since each cardiometabolic disease may share possible risk factors and reciprocal associations [34], further studies that adjust for potential mutual confounders are needed [16].

Therefore, this study aimed to compare the presence of environmental influences on the genetic portion of cardiometabolic diseases using twin cohorts. We investigated the frequency and concordance of cardiometabolic diseases and the absolute differences in laboratory parameters contributing to cardiometabolic conditions between monozygotic twins (genetic influence) and dizygotic twin pairs (environmental contribution over genetic influence) after adjusting for lifestyle factors to explore this issue.

## 2. Materials and Methods

### 2.1. Study Population and Data Collection

The Ethics Committee of Hallym University approved this study (2021-03-004). The Institutional Review Board exempted the requirement for written informed consent. This cohort study used data from 2005 through 2014 from the Korean Genome and Epidemiology Study (KoGES, http://www.nih.go.kr/NIH/eng/main.jsp) (accessed on 1 January 2020), which is a database of community-based prospective cohort studies launched in 2001 [35]. From the KoGES Consortium, we used the KoGES Healthy Twin Study (HTS) (baseline and follow-up data from 2005 to 2013 and 2008 to 2014, respectively), which is an ongoing multicenter cohort study of urban residents aged ≥ 20 years that was launched in 2005 [36]. Zygosity was assessed at baseline using a questionnaire with >90% accuracy and a genetic analysis, including 16 short tandem repeat markers (AmpFlSTR Identifier Kit; Perkin Elmer, Waltham, MA, USA) [37]. Two-thirds of the participants who underwent the baseline examination were followed up, and their medical histories were updated.

### 2.2. Participant Selection

**Figure 1:**
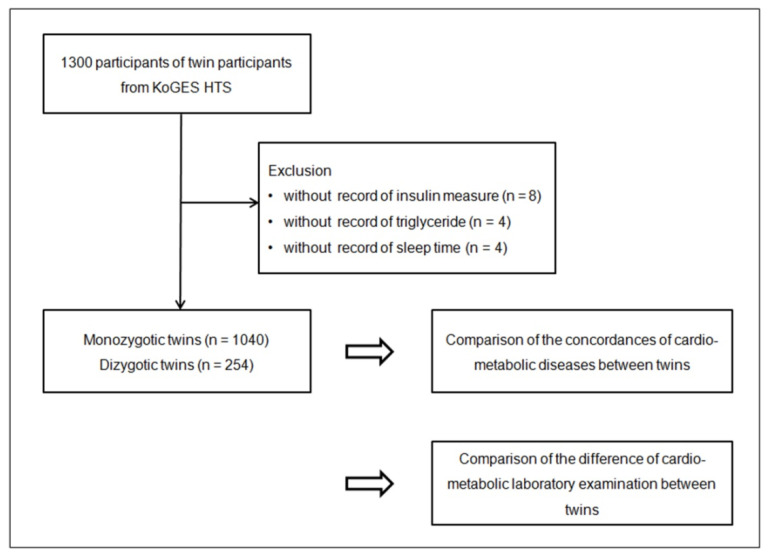
The study design of this study. The 1040 monozygotic twins and 254 dizygotic twins were compared for the concordance of various cardiometabolic diseases and the difference in physical and laboratory examination results between twins.

A total of 1300 twin participants were selected from the KoGES HTS database for this cross-sectional study. Participants without records for insulin levels (*n* = 8), triglyceride measurements (*n* = 4), and sleep time (*n* = 4) were excluded from the list. Overall, 1040 monozygotic (520 pairs of twins) and 254 dizygotic (127 pairs of twins) twin participants were enrolled (Figure 1). Subsequently, we investigated the concordance of the histories of cardiometabolic diseases and the differences in laboratory parameters between the monozygotic and dizygotic twin groups.

### 2.3. Survey

Past medical history was documented, and a physical examination was conducted. Fasting venous blood samples were obtained to measure laboratory parameters. Briefly, trained interviewers asked the attendees about their history of cardiometabolic diseases (hypertension, hyperlipidemia, type 2 diabetes mellitus, cerebral stroke, transient ischemic attack, and ischemic heart disease). Hemoglobin A1c (g/dL), low-density lipoprotein (LDL, mg/dL), high-density lipoprotein (HDL, mg/dL), triglyceride (mg/dL), total cholesterol (mg/dL), insulin (uIU/mL), and fasting blood glucose (mg/dL) levels were measured by blood sampling. Systolic blood pressure (SBP, mmHg) and diastolic blood pressure (DBP, mmHg) were also checked. Monthly income was categorized as non-respondent, low income (<$2000 per month), middle income ($2000–$3999 monthly), and high income (≥$4000 monthly) based on the household income. Educational status was classified as under-high school, high school, or college (dropped out or graduated from college). Marital status was classified as unmarried, married, divorced, or other. Physical activity levels were categorized into hard, moderate, walking time, and sitting time in either the workplace or at home. Body mass index (BMI) was expressed in kg/m^2^ using the health checkup data. Smoking history was classified as non-smoker (<100 cigarettes in entire life), past smoker (quit for more than 1 year), and current smoker. Drinking habits were grouped into nondrinkers, ≤1 time per month, 2–4 times monthly, and ≥2 times weekly. Sleep time was measured on 5/7 weekdays plus 2/7 weekends.

### 2.4. Exposure

Twin types (monozygotic and dizygotic) were considered independent variables in this study. Participants with multiple births other than twins were excluded.

### 2.5. Outcome 

We estimated the concordance of cardiometabolic diseases between matched twin participants, as the primary measurement in a classical twin study is the concordance rate; a higher concordance between monozygotic twin pairs than between dizygotic ones may be considered to indicate a genetic contribution over environmental factors, whereas a similar degree of difference between monozygotic and dizygotic twin groups may be considered attributed to an environmental influence. These were sub-grouped as positive-positive, positive-negative, or negative-negative. If both twin siblings had or never had the same disease or trait, they were considered concordant (positive-positive and negative-negative, respectively). Additionally, we calculated the absolute differences in laboratory examination data and blood pressure between the matched twin participants. For example, if one of the twin participants had an SBP of 140 mmHg and the other showed an SBP of 130 mmHg, the absolute difference in SBP was 10 mmHg.

### 2.6. Statistical Analyses

A chi-square test (categorical variables) or Wilcoxon rank-sum test (continuous variables) was conducted to compare the baseline features of the participants. We calculated odds ratios (OR) with 95% confidence intervals (CI) for the concordance of cardiometabolic diseases. First, we calculated the OR of monozygotic twins ([positive-positive or negative-negative]/[positive-negative]) compared with that of dizygotic twins using a binomial logistic regression model. Second, we calculated the OR of monozygotic twins ([positive-positive]/[positive-negative]/[negative-negative]/) compared with that of dizygotic twins using a multinomial logistic regression model. 

We determined the estimated values (EVs) with their 95% CI of the absolute difference between laboratory examination and blood pressure. EV was assessed as the “absolute difference between monozygotic twins” minus the “absolute difference between dizygotic twins” by applying a linear regression model. 

Crude, adjusted model 1 (age, sex, income, education, marital status, physical activity, obesity, smoking habit, frequency of alcohol consumption, and sleep time) and adjusted model 2 (model 1 plus history of each disease [hypertension, hyperlipidemia, diabetes mellitus, cerebral stroke, transient ischemic attack, and myocardial infarction]) were used to evaluate the findings. Furthermore, two-tailed analyses were conducted, and statistical significance was set at *p* < 0.05. The results were analyzed using the statistical package for the social sciences (version 24.0; IBM, Armonk, NY, USA).

## 3. Results

Comparisons in the baseline characteristics of monozygotic and dizygotic twins are presented in Table 1. The rates of cardiometabolic diseases, including hypertension, hyperlipidemia, type 2 diabetes, cerebral stroke, transient ischemic attack, or ischemic heart disease, and their related physical and laboratory examination levels were not significantly different in monozygotic and dizygotic twins (all *p* > 0.05). However, the distribution of age groups, sex ratio, and hard physical activity level differed between monozygotic and dizygotic twins (*p* = 0.004, *p* = 0.024, and *p* = 0.015, respectively). Other variables, including income level, education level, marital status, other physical activity levels (except for the hard level), obesity, smoking status, frequency of alcohol consumption, and sleep hours, were similar between the two groups (all *p* > 0.05).

We examined the concordance rates regarding the presence or absence of cardiometabolic diseases in monozygotic twins compared with dizygotic twins (Table 2). Although the crude ORs for hypertension were significantly different in the concordance rates of monozygotic twins (1.53; 95% CI = 1.02–2.29; *p* = 0.042), the fully adjusted analysis revealed no significant associations (1.42; 95% CI = 0.88–2.29; *p* = 0.155). The adjusted ORs for the concordance rates of hyperlipidemia, type 2 diabetes, cerebral stroke, transient ischemic attack, and ischemic heart disease were not significantly different between monozygotic and dizygotic twins (all *p* > 0.05). The odds of concordance for cardiometabolic diseases in monozygotic twins were not significantly higher than those in dizygotic twins.

Next, we investigated whether at least one incident of cardiometabolic disease was more frequent in monozygotic twin pairs or dizygotic twin pairs (Table 3). In the crude ORs and adjusted ORs, the incidence of cardiometabolic diseases within twins was not statistically higher (all *p* > 0.05).

The differences in laboratory exam levels related to cardiometabolic diseases between the matched monozygotic and dizygotic twin pairs were calculated (Table 4). The differences in the levels of hemoglobin A1c (EV = 0.14, 95% CI = 0.03–0.026, *p* = 0.011), total cholesterol (EV = 7.73, 95% CI = 4.82–10.65, *p* < 0.001), HDL-cholesterol (EV = 3.38, 95% CI = 2.44–4.32, *p* < 0.001), LDL-cholesterol (EV = 5.68, 95% CI = 3.04–8.32, *p* < 0.001), triglycerides (EV = 9.84, 95% CI = 1.67–18.02, *p* = 0.018), fasting insulin (EV = 0.97, 95% CI = 0.56–1.37, *p* < 0.001), and SBP (EV = 2.39, 95% CI = 1.05–3.72, *p* < 0.001) were more remarkable within dizygotic twins than within monozygotic twins, suggesting a more similar tendency in monozygotic twins.

## 4. Discussion

In this cross-sectional study of validated twin cohorts, we could not demonstrate any significant concordance between cardiometabolic diseases or any increased likelihood of cardiometabolic diseases in monozygotic and dizygotic twins. Nevertheless, most serological and physical parameters related to cardiometabolic diseases indicated a more comparable tendency within monozygotic twin pairs than in dizygotic ones. Because of the rarity of validated twin cohort data, our current epidemiological study contributes to the existing understanding of the influence of environmental factors over the genetic contribution in overt cardiometabolic diseases through concurrent analyses of cardiometabolic risk factors.

The concordance in the occurrence of hypertension, hyperlipidemia, type 2 diabetes, cerebral stroke, transient ischemic attack, or ischemic heart disease in monozygotic twin pairs was not higher than that in dizygotic twins in this study, which might be attributed to the possible contribution of environmental factors relevant to dissimilar acquired lifestyle behaviors in monozygotic twins who share an identical genetic background, sex, and age [19]. In the Japanese monozygotic study reared separately, the concordance rate on blood pressure was 51% at the age of separation 0–5 years and 78% at the age of separation of 26 years and over, indicating the importance of the shared environmental factors in the blood pressure [38]. In coronary heart disease, the magnitude of the relative hazard of mortality from coronary heart disease decreases with age in either monozygotic or dizygotic twins, which suggests a relative increase in environmental effects at older ages [18]. In diabetes, a 58% concordance rate between monozygotic twins was observed, implying that non-genetic factors may also influence diabetes development [39]. The discordance in phenotypic disease expression between monozygotic twin pairs may also be partly explained by epigenetic differences [40]. Although twins are epigenetically indistinct during the early years of life, older monozygotic twins have prominent disparities in their genomic aspects of 5-methylcytosine DNA and histone acetylation [41], influencing differential gene expression signatures and disease phenotypes with age [29]. Meta-analyses and twin studies have suggested genetic and environmental interaction that possible cumulative effects of lifestyle exposures, such as physical activity and smoking, may modify genetic susceptibility to a subset of cardiometabolic diseases [42,43]. Since exclusively adult populations were included in this study, different environmental or acquired influences might mitigate the concordance likelihood of overt cardiometabolic diseases in monozygotic twin pairs compared with those in dizygotic twin pairs. 

Although genetic alterations and environmental impacts have been implicated in many cardiometabolic diseases [17], the clinical significance of baseline laboratory parameters in possible genetic and environmental effects appears to be underestimated. Despite the limited link between disease presentation and monozygotic twins, several laboratory parameters related to cardiometabolic diseases have been associated with more comparable trends within the monozygotic twin pairs than in dizygotic twins. We also found similar levels of hemoglobin A1c, LDL-cholesterol, HDL-cholesterol, total cholesterol, insulin, triglyceride, and SBP in monozygotic twin pairs, while the other parameters (glucose and DBP) showed disparity similar to that of dizygotic twins; the lesser differences of those levels in monozygotic twins than in dizygotic twins seem likely to be due to the potential contribution of heritable elements, which may partly correspond to the findings of previous studies [23,24,44]. The monozygotic twin study reared apart or together have also shown moderate to high heritability of plasma cholesterol, triglyceride concentrations, waist circumference, plasma glucose and insulin, and blood pressures [44], suggesting the dominant genetic susceptibility to those plasma and physical, metabolic abnormalities. Similarly, in a Chinese twin study, LDL-cholesterol, total cholesterol, and triglyceride levels were remarkably affected by genetic factors [32]. However, one Hungarian twin study reported a potential inherited predisposition to SBP and DBP and a higher likelihood of an environmental influence on HDL-cholesterol, total cholesterol, triglyceride, fasting blood glucose, and insulin levels [30], which was in contrast to our findings. The heritability estimates for SBP variably range from 13% to 82%, and those for DBP range from less than 1% to 64%, with average levels of approximately 50% for both [25,45]. This discrepancy might be due to the higher ratio of female twins, smaller numbers of monozygotic and dizygotic twins, and less comprehensive adjustment of confounding factors than our study, potentially indicating the need for caution while interpreting the findings of twin studies [19].

The strength of the current study was the use of prospective twin cohort data with follow-up data for both monozygotic and dizygotic twins, KoGES HTS, and qualified data quality with regular validation by national statisticians, which made our findings more reliable. In addition, we comprehensively considered potential confounders among lifestyle factors, including obesity, physical activity, smoking, sleep duration, alcohol consumption, and socioeconomic factors, including income level, education level, and marital status, for comparisons between twin pairs. Adjustments in the abovementioned lifestyle factors may be additional strengths because these factors have been documented as relevant risk factors for cardiometabolic diseases [9].

This study had some limitations that should be addressed. First, although many variables were adjusted in the current study, unmeasured confounders could not be completely excluded. Second, owing to the cross-sectional study design, the causal relationship between twin birth and cardiometabolic diseases could not be confirmed. Third, the small number of twin pairs with cardiometabolic diseases may have limited the generalizability of the study results, even though our study represents the largest sample of twin participants. Fourth, the lack of genetic data on related cardiometabolic diseases or information on a diet may be another limitation.

## 5. Conclusions

While several baseline laboratory parameters involved in cardiometabolic disease showed a strong likelihood of heritability between monozygotic twins, the cardiometabolic disease phenotype is probably affected by environmental factors. Our results provide further supportive evidence for the preventability of cardiometabolic diseases.

## Figures and Tables

**Table 1:** Baseline characteristics of monozygotic and dizygotic twins.

Characteristics	Total Participants		
	Monozygotic Twin	Dizygotic Twin	*p*
Age (years old, *n*, %)			0.004 *
20–24	6 (0.6)	0 (0.0)	
25–29	68 (6.5)	4 (1.6)	
30–34	352 (33.8)	87 (35.7)	
35–39	244 (23.5)	65 (26.6)	
40–44	139 (13.4)	36 (14.8)	
45–49	131 (12.6)	20 (8.2)	
50–54	80 (7.7)	22 (9.0)	
55–59	14 (1.3)	10 (4.1)	
60–64	4 (0.4)	0 (0.0)	
65+	2 (0.2)	0 (0.0)	
Sex (*n*, %)			0.024 *
Males	386 (37.1)	110 (45.1)	
Females	654 (62.9)	134 (54.9)	
Income (*n*, %)			0.983
<2 million (won)	346 (33.3)	81 (33.2)	
2 to <3 million (won)	282 (27.1)	68 (27.9)	
3 to <4 million (won)	209 (20.1)	50 (20.5)	
≥4 million (won)	203 (19.5)	45 (18.4)	
Education (*n*, %)			0.743
Under high school	121 (11.6)	25 (10.2)	
Graduated from High school	366 (35.2)	92 (37.7)	
Commercial college-Dropped out of college	123 (11.8)	32 (13.1)	
Graduated from college	430 (41.3)	95 (38.9)	
Marriage (*n*, %)			0.26
Unmarried	240 (23.1)	50 (20.5)	
Married	733 (70.5)	173 (70.9)	
Divorced or others	67 (6.4)	21 (8.6)	
Physical Activity			
Hard (hour/1 week, mean, SD)	3.1 (6.8)	4.7 (9.7)	0.015 *
Moderate (hour/1 week, mean, SD)	5.9 (10.5)	6.2 (10.2)	0.651
Walk (hour/1 week, mean, SD)	6.2 (9.6)	6.8 (10.9)	0.339
Sit (hour/1 week, mean, SD)	39.9 (21.9)	37.9 (20.7)	0.190
Obesity (*n*, %)			0.241
Underweight (BMI < 18.5)	27 (2.6)	5 (2)	
Normal (BMI ≥ 18.5 to <23)	499 (48)	113 (46.3)	
Overweight (BMI 23 to <25)	220 (21.2)	68 (27.9)	
Obese I (BMI ≥ 25 to <30)	262 (25.2)	52 (21.3)	
Obese II (BMI ≥ 30)	32 (3.1)	6 (2.5)	
Smoking status (*n*, %)			0.180
Nonsmoker	680 (65.4)	145 (59.4)	
Past smoker	108 (10.4)	33 (13.5)	
Current smoker	252 (24.2)	66 (27)	
Frequency of drinking alcohol (*n*, %)			0.249
Nondrinker	301 (28.9)	64 (26.2)	
≤1 time monthly	230 (22.1)	46 (18.9)	
2–4 times monthly	300 (28.8)	80 (32.8)	
≥2 times weekly	209 (20.1)	54 (22.1)	
Sleeping hours (*n*, %)			0.370
≤5 h	53 (5.1)	16 (6.6)	
6–7 h	610 (58.7)	146 (59.8)	
8–9 h	349 (33.6)	72 (29.5)	
≥10 h	28 (2.7)	10 (4.1)	
Cardio-metabolic diseases (categorical)			
Hypertension (*n*, %)	95 (9.1)	22 (9.0)	1.000
Hyperlipidemia (*n*, %)	75 (7.2)	14 (5.7)	0.485
Type 2 diabetes mellitus (*n*, %)	37 (3.6)	7 (2.9)	0.699
Cerebral stroke (*n*, %)	6 (0.6)	1 (0.4)	1.000
Transient Ischemic Attack (*n*, %)	1 (0.1)	1 (0.4)	0.344
Ischemic heart disease (*n*, %)	15 (1.4)	4 (1.6)	1.000
Physical and laboratory examination (continuous)			
Hemoglobin A1c (g/dL, mean, SD)	13.9 (1.7)	14 (1.7)	0.493
Total cholesterol (mg/dL, mean, SD)	188.1 (33.9)	184.8 (32.9)	0.175
HDL (mg/dL, mean, SD)	51.9 (12.6)	51.4 (11.9)	0.523
LDL (mg/dL, mean, SD)	113.5 (30.5)	111.3 (30.2)	0.294
Triglyceride (mg/dL, mean, SD)	114.9 (78.4)	113.9 (82)	0.867
Insulin (uIU/mL, mean, SD)	7.5 (3.3)	7.5 (3.9)	0.994
Fasting blood glucose (mg/dL, mean, SD)	91.7 (18.8)	90.7 (12.8)	0.469
Systolic Blood Pressure (mmHg, mean, SD)	111.0 (15.6)	112.4 (14.6)	0.205
Diastolic Blood Pressure (mmHg, mean, SD)	72.0 (11.5)	71.8 (10.9)	0.747

* Significance at *p* < 0.05. A chi-square test (categorical variables) or Wilcoxon rank-sum test (continuous variables) was performed. BMI, body mass index; LDL, low-density lipoprotein; HDL, high-density lipoprotein.

**Table 2:** Analysis of odds ratios with 95% confidence interval of coincidence of cardio-metabolic diseases of monozygotic twin compared to dizygotic twin (reference: positive/negative of diseases between twin).

Coincidence of Diseases	Monozygotic Twin	Dizygotic Twin	Odds Ratios (95% Confidence Interval)					
	*n* (%)	*n* (%)	Crude	*p*	Model 1 †	*p*	Model 2 ‡	*p*
Hypertension								
concordant	934/1040 (89.8)	208/244 (85.2)	1.53 (1.02–2.29)	0.042 *	1.33 (0.84–2.11)	0.229	1.42 (0.88–2.29)	0.155
discordant	106/1040 (10.2)	36/244 (14.8)	1		1		1	
Hyperlipidemia								
concordant	956/1040 (91.9)	216/244 (88.5)	1.48 (0.94–2.32)	0.092	1.52 (0.92–2.52)	0.103	1.55 (0.93–2.59)	0.097
discordant	84/1040 (8.1)	28/244 (11.5)	1		1		1	
Type 2 diabetes								
concordant	1002/1040 (96.3)	230/244 (94.3)	1.61 (0.86–3.01)	0.141	1.66 (0.82–3.39)	0.162	1.63 (0.79–3.36)	0.183
discordant	38/1040 (3.7)	14/244 (5.7)	1		1		1	
Cerebral stroke								
concordant	1028/1040 (98.8)	242/244 (99.2)	0.71(0.16–3.18)	0.653	0.84 (0.13–5.41)	0.852	0.60 (0.06–6.11)	0.667
discordant	12/1040 (1.2)	2/244 (0.8)	1		1		1	
Transient ischemic attack								
concordant	1038/1040 (99.8)	242/244 (99.2)	4.29 (0.60–30.60)	0.146	N/A	0.977	N/A	0.988
discordant	2/1040 (0.2)	2/244 (0.8)	1		1		1	
Ischemic heart disease								
concordant	1014/1040 (97.5)	240/244 (98.4)	0.65 (0.23–1.80)	0.427	0.63 (0.19–2.04)	0.438	0.75 (0.22–2.52)	0.639
discordant	26/1040 (2.5)	4/244 (1.6)	1		1		1	

† Adjusted for age, sex, income, education, marital status, physical activity, obesity, smoking habit, frequency of drinking alcohol, and sleep time. ‡ Model 1 plus histories of each disease (hypertension, hyperlipidemia, type 2 diabetes, cerebral stroke, transient ischemic attack, and myocardial infarction). “Concordant” means concordant positive-positive or negative-negative results between monozygotic twins or between dizygotic twins, whereas “discordant” means discordant positive and negative results between monozygotic twins or between dizygotic twins. * *p* < 0.05.

**Table 3:** Analysis of odds ratios with 95% confidence interval of occurrence of at least one cardio-metabolic disease of monozygotic twin compared to dizygotic twin (reference: negative/negative of diseases between twin).

Coincidence of Diseases	Monozygotic Twin	Dizygotic Twin	Odds Ratios (95% CI)					
	*n* (%)	*n* (%)	Crude	*p*	Model 1 †	*p*	Model 2 ‡	*p*
Hypertension								
Positive-positive	42/1040 (4)	4/244 (1.6)	2.40 (0.85–6.77)	0.098	3.61 (1.16–11.21)	0.026 *	2.13 (0.83–5.45)	0.114
Positive-negative	106/1040 (10.2)	36/244 (14.8)	0.673 (0.45–1.01)	0.057	0.76 (0.48–1.21)	0.249	0.72 (0.45–1.16)	0.180
Negative-negative	892/1040 (85.8)	204/244 (83.6)	1		1		1	
Hyperlipidemia								
Positive-positive	32/1040 (3.1)	0/244 (0)	N/A	N/A	N/A	N/A	N/A	N/A
Positive-negative	84/1040 (8.1)	28/244 (11.5)	0.70 (0.45–1.10)	0.124	0.71 (0.43–1.15)	0.164	0.69 (0.43–1.10)	0.121
Negative-negative	924/1040 (88.8)	216/244 (88.5)	1		1		1	
Type 2 diabetes								
Positive-positive	18/1040 (1.7)	0/244 (0)	N/A	N/A	N/A	N/A	N/A	N/A
Positive-negative	38/1040 (3.7)	14/244 (5.7)	0.63 (0.34–1.19)	0.156	0.66 (0.33–1.30)	0.228	0.65 (0.33–1.30)	0.223
Negative-negative	984/1040 (94.6)	230/244 (94.3)	1		1		1	
Cerebral stroke								
Positive-positive	0/1040 (0)	0/244 (0)	N/A	N/A	N/A	N/A	N/A	N/A
Positive-negative	12/1040 (1.2)	2/244 (0.8)	1.41 (0.31–6.35)	0.653	1.59 (0.33–7.75)	0.567	1.40 (0.28–7.04)	0.680
Negative-negative	1028/1040 (98.8)	242/244 (99.2)	1		1		1	
Transient ischemic attack								
Positive-positive	0/1040 (0)	0/244 (0)	N/A	N/A	N/A	N/A	N/A	N/A
Positive-negative	2/1040 (0.2)	2/244 (0.8)	0.23 (0.03–1.66)	0.146	0.31 (0.04–2.60)	0.278	0.04 (0.00–2.32)	0.135
Negative-negative	1038/1040 (99.8)	242/244 (99.2)	1		1		1	
Ischemic heart diseases								
Positive-positive	2/1040 (0.2)	2/244 (0.8)	0.24 (0.03–1.68)	0.149	0.01 (0.00–1.50)	0.074	0.62 (0.05–7.70)	0.710
Positive-negative	26/1040 (2.5)	4/244 (1.6)	1.53 (0.53–4.42)	0.434	1.60 (0.53–4.84)	0.410	1.15 (0.38–3.48)	0.809
Negative-negative	1012/1040 (97.3)	238/244 (97.5)	1		1		1	

† Adjusted for age, sex, income, education, marital status, physical activity, obesity, smoking habit, frequency of drinking alcohol, and sleep time. ‡ Model 1 plus histories of each disease (hypertension, hyperlipidemia, type 2 diabetes, cerebral stroke, transient ischemic attack, and myocardial infarction). * *p* < 0.05.

**Table 4:** Analysis of estimated values of the absolute value of the difference between the matched twins (reference: the absolute value of the difference between monozygotic twins).

Differences in Clinical Examination	Estimated Values of the Absolute Difference between Twins (95% CI)					
	Crude	*p*	Model 1 †‡	*p*	Model 2 ‡	*p*
Differences in Hemoglobin A_1c_	0.12 (0.01–0.23)	0.030 *	0.14 (0.03–0.25)	0.011 *	0.14 (0.03–0.26)	0.011 *
Differences in Total Cholesterol	7.45 (4.49–10.42)	<0.001 *	7.41 (4.45–10.37)	<0.001 *	7.73 (4.82–10.65)	<0.001 *
Differences in HDL-Cholesterol	3.44 (2.50–4.37)	<0.001 *	3.36 (2.42–4.29)	<0.001 *	3.38 (2.44–4.32)	<0.001 *
Differences in LDL-Cholesterol	5.50 (2.80–8.20)	<0.001 *	5.42 (2.73–8.11)	<0.001 *	5.68 (3.04–8.32)	<0.001 *
Differences in Triglyceride	10.38 (1.80–18.95)	0.018 *	9.13 (0.94–17.31)	0.029 *	9.84 (1.67–18.02)	0.018 *
Differences in Insulin	0.91 (0.50–1.31)	<0.001 *	0.96 (0.55–1.36)	<0.001 *	0.97 (0.56–1.37)	<0.001 *
Differences in Glucose	0.55 (−1.54–2.65)	0.604	0.14 (−1.94–2.21)	0.897	0.33 (−1.70–2.37)	0.749
Differences in SBP	2.40 (1.03–3.77)	0.001 *	2.39 (1.01–3.77)	0.001 *	2.39 (1.05–3.72)	<0.001 *
Differences in DBP	0.83 (−0.24–1.90)	0.130	0.65 (−0.43–1.73)	0.241	0.90 (−0.17–1.96)	0.098

† Adjusted for age, sex, income, education, marital status, physical activity, obesity, smoking habit, frequency of drinking alcohol, and sleep time. ‡ Model 1 plus histories of each disease (hypertension, hyperlipidemia, type 2 diabetes, cerebral stroke, transient ischemic attack, and myocardial infarction). BMI, body mass index; LDL, low-density lipoprotein; HDL, high-density lipoprotein; SBP, systolic blood pressure; DBP, diastolic blood pressure. * *p* < 0.05.

## Data Availability

Restrictions apply to the availability of these data. Data were obtained from the Korean Genome and Epidemiology Study (KoGES) and are available at https://www.nih.go.kr/contents.es?mid=a50401010100#1 (accessed on 1 January 2022).
